# Early hepatoid adenocarcinoma of the stomach with signet ring cell carcinoma: A case report and clinicopathological features

**DOI:** 10.3389/fonc.2022.1016447

**Published:** 2023-01-11

**Authors:** Xinglong Wu, Lianjun Di, Chengfang Li, Suyuan Zhang, Na Tan, Jiajia Huang, Biguang Tuo

**Affiliations:** ^1^ Department of Pathology, Affiliated Hospital of Zunyi Medical University, Zunyi, China; ^2^ Department of Gastroenterology, Digestive Disease Hospital, Affiliated Hospital of Zunyi Medical University, Zunyi, China

**Keywords:** case report, clinicopathological feature, hepatoid adenocarcinoma, signet ring cell carcinoma, stomach

## Abstract

**Background:**

Hepatoid adenocarcinoma of the stomach (HAS) is a rare subtype of gastric cancer with poor prognosis, and its clinicopathological features are not well understood, so the pathology from the clinical biopsy is easily misdiagnosed, especially for special or atypical HAS. We present an extremely rare early HAS with signet ring cell carcinoma and evaluate its clinicopathological features.

**Case presentation:**

A 51-year-old female patient of Chinese Han ethnicity with upper abdominal pain for 5 years and worsened abdominal pain for 1 month was admitted to our hospital. Esophagogastroduodenoscopy showed a submucosal tumor-like elevated lesion with central depression in the greater curvature of the junction between the antrum and body. Histopathological examination from the biopsy revealed medium–low-differentiation adenocarcinoma with signet ring cell carcinoma. Radical gastrectomy was performed, and the final diagnosis was early HAS with signet ring cell carcinoma.

**Conclusions:**

HAS with signet ring cell carcinoma is a special type of HAS and extremely rare. It is first presented for this extremely rare type of HAS, which contributes to strengthen the understanding for the clinicopathological characteristics of HAS and especially promote early detection of HAS.

## Introduction

Hepatoid adenocarcinoma of the stomach (HAS) refers to a special type of gastric cancer with the characteristics of adenocarcinoma and hepatocarcinoid differentiation originating in the gastric mucosa with or without the increase of serum alpha fetoprotein (AFP). It is a rare form of gastric cancer, accounting for 0.3%–1% of all gastric cancers (1, 2). According to a literature search of PubMed/Medline, approximately 500 cases of HAS have been reported in the world, mainly in case reports and clinical or pathological analyses, but little is about the report of early HAS (3). HAS progresses rapidly, especially with high potential for liver and lymph node metastasis, and the prognosis is very poor (4, 5). The symptoms of patients with HAS are similar to those of common gastric cancer, and the diagnosis of HAS is mainly dependent on the endoscopy and pathological analysis. However, it is sometimes difficult to do pathological diagnosis by gastroscopic biopsy and is easily misdiagnosed. Thus, the challenge remains in the appropriate diagnosis of this rare entity, especially early diagnosis, to improve its unfavorable prognosis. We herein present an extremely rare early HAS with signet ring cell carcinoma and analyze its clinicopathological features that contribute to the improvement of the diagnosis of HAS.

## Case presentation

**Figure 1:**
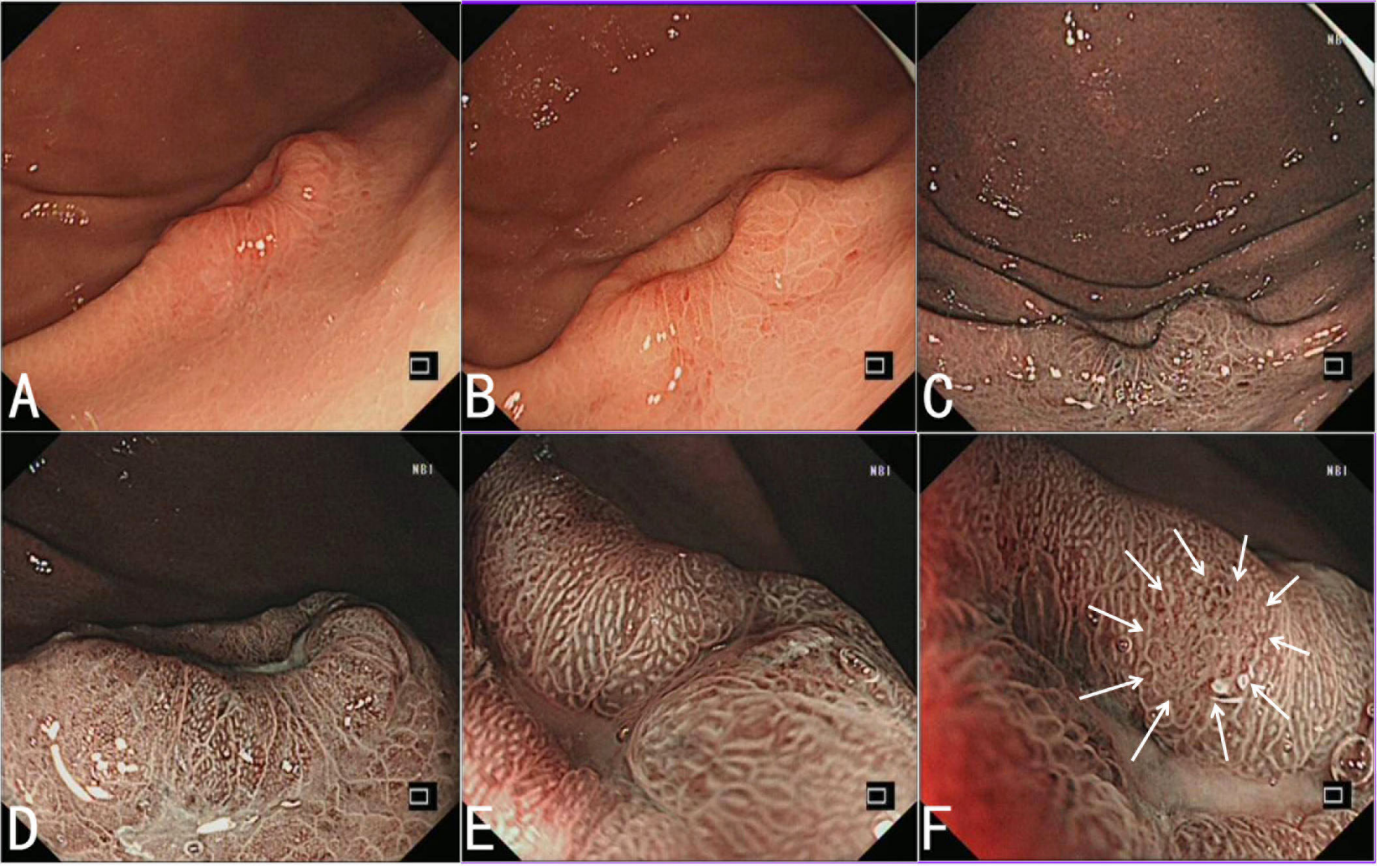
Endoscopic features of HAS. **(A, B)** White light endoscopy shows a 20-mm type 0–IIa+IIc lesion with submucosal tumor-like change in the greater curvature of the junction between the antrum and body. The lesion has a sense of compactness and fullness with a central depression. **(C)** NBI shows a brownish area with an indistinct boundary. **(D–F)** ME-NBI with low **(D)** and high **(E)** magnification shows that most parts of the lesion present as a regular microvascular pattern, and there is the presence of a demarcation line, some widened intervening parts, and elongated pits, and only small parts present as an irregular microvascular pattern (indicated by the arrow) **(F)**.

A 51-year-old female patient of Chinese Han ethnicity was admitted to our hospital because of upper abdominal pain for 5 years and worsened abdominal pain for 1 month. There was no significant weight loss and special past medical history. Physical examination had no abnormal findings. Esophagogastroduodenoscopy (EGD) was performed. Under white light, the antrum and corpus presented as non-atrophic gastritis, without a sign of *Helicobacter pylori* infection. A 20-mm type 0–IIa+IIc lesion with submucosal tumor-like change in the greater curvature of the junction between the antrum and body was detected. The lesion has a sense of compactness and fullness with a central depression (**Figures 1A, B**). Narrow-band imaging (NBI) showed a brownish area with an indistinct boundary (**Figure 1C**). Magnified endoscopy with NBI (ME-NBI) at low and high magnification revealed that most parts of the lesion presented as a regular microvascular pattern and only small parts presented as an irregular microvascular pattern, and there was the presence of a demarcation line, widened intervening parts, and elongated pits (**Figures 1D–F**). It was considered as an undifferentiated cancer, lymphoepithelioid cancer, or other special type of tumor by endoscopy. The pathological examination from biopsy revealed that the lesion was medium–low-differentiation adenocarcinoma with signet ring cell carcinoma.

Abdominal computed tomography revealed a local inhomogeneous thickening of the gastric wall at the greater curvature of the gastric body, calcification of the right lobe of the liver, uterine fibroids, and right ovarian cyst. Serum tumor-associated antigen and tumor marker test showed that α-L-fucosidase, carcinoembryonic antigen, carbohydrate antigen 19-9, carbohydrate antigen 153, carbohydrate antigen 125, ferritin, AFP, and human chorionic gonadotropin (hCG) were all normal.

Radical distal gastrectomy was performed for the patient. Surgery examination showed that the lesion was located in the greater curvature of the junction between the antrum and body, with a size of approximately 20 mm × 12 mm. The lesion did not break through the serous membrane and had no obvious adhesion with the surrounding tissues. A total of 20 lymph nodes in the greater curvature and nine in the lesser curvature of the stomach were palpated. There was no obvious metastasis in the liver, gallbladder, spleen, bilateral kidneys, transverse colon, mesentery, abdominal wall, and pelvic cavity.

**Figure 2:**
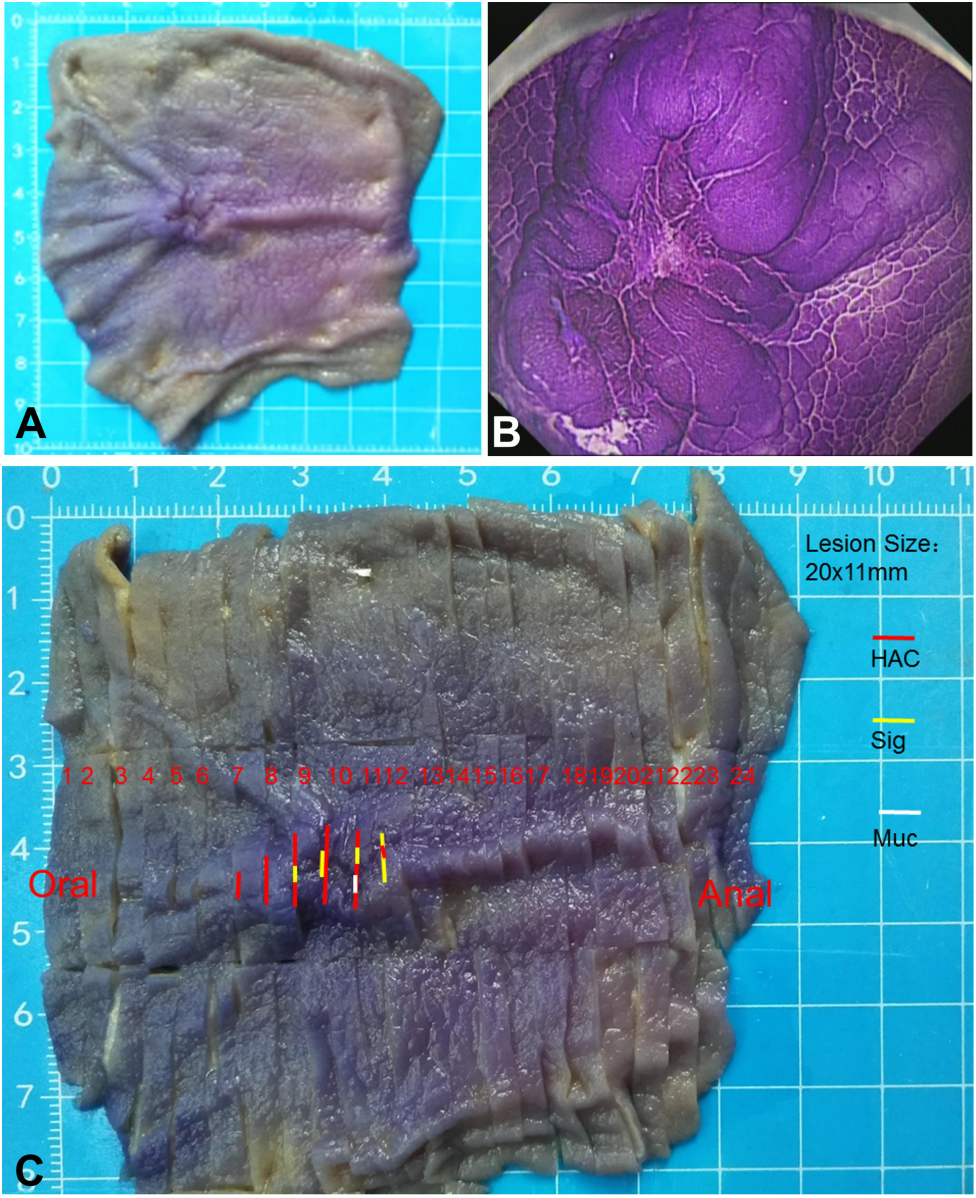
Macroscopic morphology of the resected specimen. **(A)** Macroscopic observation shows a protuberant lesion with a central depression. The surrounding mucosa is smooth, with the converging of the mucosal folds toward the lesion and the fusion of folds. **(B)** Crystal violet staining of the resected specimen more clearly shows the morphology of the lesion. **(C)** The lesion and its surrounding tissues are taken every 2–3 mm for histopathological examination according to the cutting standard of early gastric cancer specimens. HAC (red line), representing the areas with hepatoid adenocarcinoma of the stomach; Sig (yellow line), representing the areas with signet ring cell carcinoma; Muc (white line), representing the areas with the formation of mucin.

Examination of the resected specimen revealed that there was a mucosal protuberant lesion with a central depression in the greater curvature of the junction between the antrum and body, and the size was 20 mm × 11 mm. The surrounding mucosa was smooth, with converging of the mucosal folds toward the lesion and fusion of the folds (**Figure 2A**). The crystal violet staining for the resected specimen more clearly revealed the morphology of the lesion (**Figure 2B**). The lesion and its surrounding tissues were taken every 2–3 mm for histopathological examination according to the cutting standard of early gastric cancer specimens (**Figure 2C**). The lymph nodes were also taken for histopathological examination.

**Figure 3:**
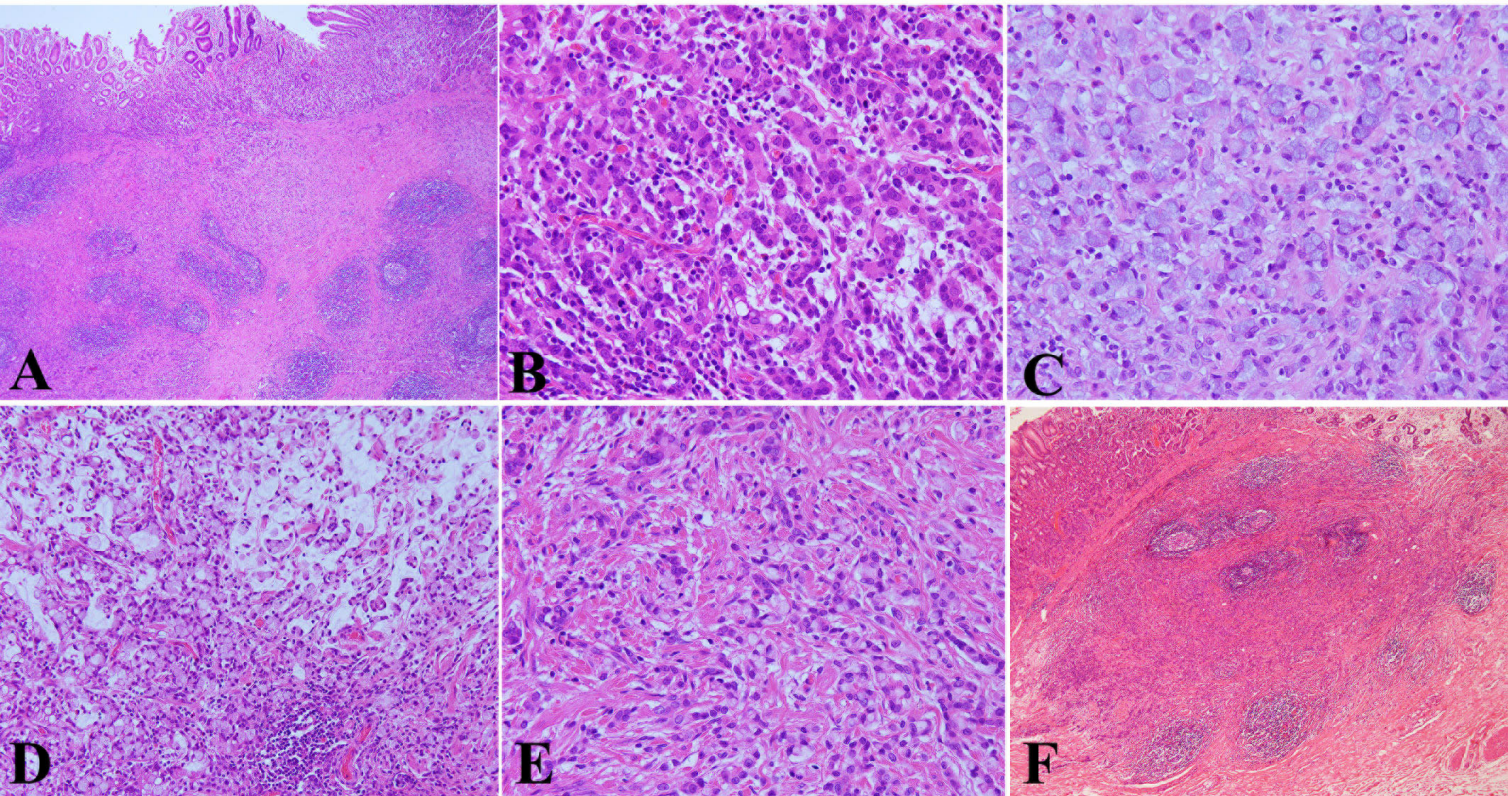
Pathological features of HAS by hematoxylin & eosin (HE) staining. **(A)** The infiltration of cancer tissue was limited to the mucosa and submucosa and reached the deep layer of the submucosa. The mucosal surface was almost covered by non-neoplastic epithelium (HE, ×40 magnification). **(B)** Some cancer cells were arranged in the form of irregular nests, cords, or micro glandular tubes. The cells were large and cubic in size, rich and eosinophilic in the cytoplasm, and with round or oval nucleus, binucleate and small nucleoli, and hepatocyte-like appearance (HE, ×400 magnification). **(C)** Some cancer cells displayed the form of a signet ring and distributed diffusely (HE, ×400 magnification). **(D)** There was mucus formation in the interstitium of some signet ring cells, the signet ring cells floated in mucus (HE, ×200 magnification). **(E)** The different forms of cancer cells were interlaced with each other (HE, ×400 magnification). **(F)** In the submucosa, the stromal fibrous tissue of the cancer obviously proliferated to form a nodular shape with a clear boundary, and the lymphocyte focally infiltrated and formed lymphoid follicles (HE, ×40 magnification).

Microscopically, the infiltration of cancer tissue was limited to the mucosa and submucosa and reached the deep layer of the submucosa. The mucosal surface was almost covered by non-neoplastic epithelium (**Figure 3A**). The tumor cells exhibited a different pattern. Some cancer cells were arranged in the form of irregular nests, cords, or micro glandular tubes. The cells were large and cubic in size, rich and eosinophilic in the cytoplasm, and with round or oval nucleus, binucleate and small nucleoli, and hepatocyte-like appearance (**Figure 3B**). Some cancer cells displayed the form of signet ring and distributed diffusely (**Figure 3C**). There was mucus formation in the interstitium of some signet ring cells, and the signet ring cells floated in mucus (**Figure 3D**). The different forms of cancer cells were interlaced with each other (**Figure 3E**). In the submucosa, the stromal fibrous tissue of the cancer obviously proliferated to form a nodular shape with a clear boundary, and the lymphocyte focally infiltrated and formed lymphoid follicles (**Figures 3A, F**). The gastric mucosa around the cancer exhibited mild chronic non-atrophic gastritis. No cancer metastasis was found in the 20 lymph nodes of the greater curvature and nine lymph nodes of the lesser curvature of the stomach.

**Figure 4:**
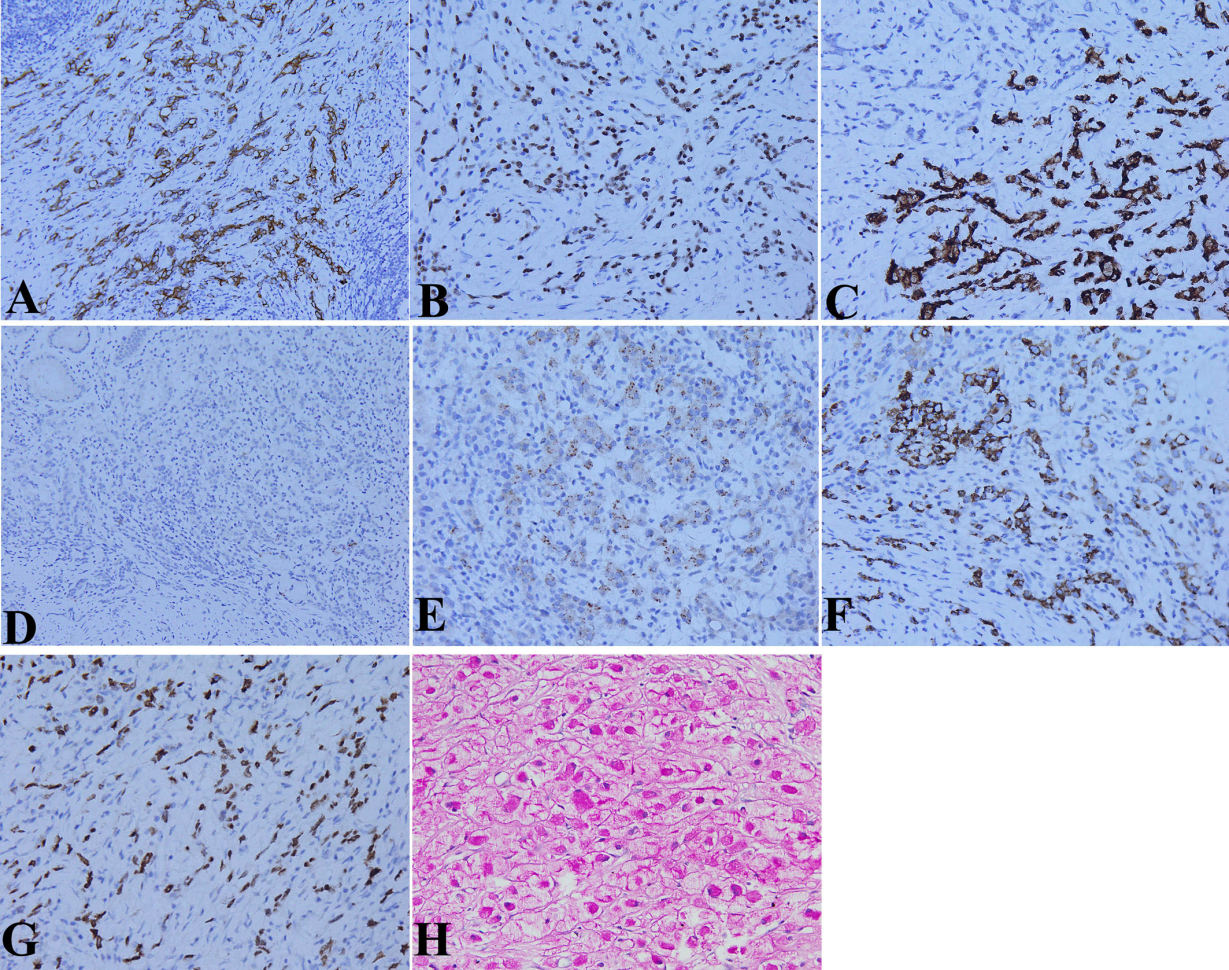
Molecular pathological features of HAS by immunohistochemical **(IHC)** staining and special staining. **(A)** Cytokeratin (CK) expression in the cytoplasm of two kinds of tumor cells (IHC staining, ×200 magnification). **(B)** Caudal-related homeobox transcription factor 2 (CDX2) expression in the nucleus of the two kinds of tumor cells (IHC staining, ×200 magnification). **(C)** Mucin 2 expression in the cytoplasm of the two kinds of tumor cells (IHC staining, ×400 magnification). **(D)** No alpha fetoprotein (AFP) expression in the cytoplasm of the two kinds of tumor cells (IHC staining, ×400 magnification). **(E)** Glypican-3 expression in the cytoplasm of hepatoid carcinoma cells (IHC staining, ×400 magnification). **(F)** Hepatocyte expression in the cytoplasm of hepatoid carcinoma cells (IHC staining, ×400 magnification). **(G)** Spalt-like transcription factor 4 (SALL4) expression in the nucleus of hepatoid carcinoma cells (IHC staining, ×400 magnification). **(H)** Red periodic acid-Schiff (PAS) staining in the cytoplasms of signet ring cells (IHC staining, ×400 magnification).

Immunohistochemical examination showed that the expressions of cytokeratin (CK) (**Figure 4A**), caudal-related homeobox transcription factor 2 (CDX2) (**Figure 4B**), and mucin 2 (**Figure 4C**) were positive; human epidermal growth factor receptor 2 (HER2), mucin 5AC, AFP (**Figure 4D**), and hCG were negative; and *in situ* hybridization showed that EBV-encoded RNA (EBER) was negative in the two forms of cancer cells. While the expressions of glypican-3 (**Figure 4E**), hepatocyte (**Figure 4F**), and spalt-like transcription factor 4 (SALL4) (**Figure 4G**) were positive in the hepatoid carcinoma cells. Periodic acid-Schiff (PAS) staining showed that the cytoplasms of signet ring cells were red (**Figure 4H**). No vascular invasion was found by CD31 and D2-40 examination.

According to histomorphology and immunohistochemical phenotypes, the final diagnosis was early HAS with signet ring cell carcinoma.

## Discussion

HAS is a rare subtype of gastric cancer, originating from the gastric mucosa and exhibiting morphological features of gastric cancer and hepatoid adenocarcinoma, so it is considered to represent gastric carcinoma with hepatic differentiation and morphological similarity to hepatic cells. Since Ishikura et al. (6) first introduced HAS in 1985, HAS has been reported in individual cases in the world in the past time. The elevated serum AFP level is regarded as a significant feature of HAS (7). However, a proportion of HAS patients have not shown an increase in serum AFP level. In our case, serum AFP was normal and there was no expression of AFP in the tumor tissue either. The diagnosis of HAS is largely dependent on its histological characteristics and immunohistochemical analysis of tumor markers, regardless of its capacity to produce AFP. Similarity to AFP, glypican-3 and SALL4 are oncofetal proteins that are produced by the fetal liver (8, 9). Since there is no AFP expression in the cancer tissues of some HASs, whereas glypican-3 and SALL4 expressions are usually positive, glypican-3 and SALL4 are considered potentially more useful biomarkers of HAS than AFP (10). In our case, both glypican-3 and SALL4 were positive in the cancer tissue, there were remarkable histomorphologic features of hepatoid structure, and hepatocyte staining was positive in the cancer tissue; therefore, the diagnosis of HAS was determined. What is special in this case is that the tumor cells exhibited different patterns. In addition to features of hepatoid carcinoma and gastric adenocarcinoma, some cells displayed the form of a signet ring, with mucus formation in the interstitium of some areas, and PAS staining for the cytoplasm of the signet cell was positive. The different forms of tumor cells were intermingled with each other. In addition, the infiltration of cancer tissue was limited to the mucosa and submucosa, without vascular invasion and lymphatic metastasis. Therefore, the final diagnosis of early HAS with signet ring cell carcinoma was determined. This is an extremely rare type of HAS. To our knowledge, it is the first time that HAS with signet ring cell carcinoma is presented in the literature.

HAS is rare but has high malignancy and poor prognosis. Due to lack of specific endoscopic characteristics and finite pathological materials of the endoscopic biopsy, it is difficult to diagnose clinically and pathologically and easily misdiagnosed. In this case, EGD showed that the antrum and corpus exhibited non-atrophic gastritis without a sign of *H. pylori* infection, and there was a protuberant lesion with a central depression in the greater curvature of the junction between the antrum and body. There was an irregular microvascular pattern only in small parts of the lesion, some widened intervening parts, and elongated pits under ME-NBI. Therefore, we think that the endoscopist should pay special attention to abnormal alterations of the gastric mucosa, even in a state without a sign of atrophy and *H. pylori* infection. A previous study also showed that HAS is not related to known risk factors for developing a common gastric adenocarcinoma, *H. pylori* infection, or chronic atrophic gastritis (11). Histologically, the tumor was limited in the mucosa and submucosa, and the surface layer of the mucosa was almost covered by non-neoplastic epithelium. The histopathology of the tumor was composed of hepatoid carcinoma, gastric adenocarcinoma, and signet ring cell carcinoma, and there was mucus formation in the interstitium of some areas. The hepatoid carcinoma, adenocarcinoma, and signet ring cell carcinoma were distributed in different regions and intermingled with each other. In the regions of adenocarcinoma and signet ring cell carcinoma, the component of adenocarcinoma was less, whereas signet ring cell carcinoma was relatively more. This histopathology was easily misdiagnosed as poorly differentiated adenocarcinoma with signet ring cell carcinoma, which should be paid more attention. Judging from the histopathological features of this case of HAS, we think that it may be more appropriate to call it gastric hepatoid signet ring cell carcinoma, but it needs the accumulation and study of more cases. It should be noted that the fibroid tissue in the submucosa was obviously proliferated, even nodular, and lymphocytes focally infiltrated and formed lymphoid follicles in the fibrous interstitium.

The molecular characteristics of HAS are not completely clear. Producing AFP is regarded as a significant feature of HAS, but a proportion of HAS has not shown an increase in serum AFP and the expression of AFP in HAS tissue. AFP production is believed to be related to HAS cell component percentage in a tumor (12). In this case, there was no increase in serum AFP and AFP expression of HAS tissue, which might be related to HAS cell component percentage in this tumor. SALL4 is a novel stem cell gene and highly expressed in both the murine and human fetal liver (13, 14). SALL4 expression has also been observed in the neofetal stomach, primitive germ cell tumors, enteroblastic adenocarcinomas, yolk sac tumors, and HAS (9). Analysis of molecular features of HAS indicated that SALL4 may play an essential role in HAS carcinogenesis (15). In this case, there was a high SALL4 expression in HAS tissue. In addition, we revealed that there were the expressions of CDX2 and mucin 2 in this HAS tissue, whereas mucin 5AC was negative, indicating that the tumor cells differentiated into intestinal epithelium.

In conclusion, HAS with signet ring cell carcinoma is a special type of HAS and extremely rare. This is the first report of endoscopic and histologic presentation of the case, which contributes to strengthen the understanding on its clinicopathological characteristics and especially promote early detection to improve the patient’s outcome. Endoscopists and pathologists should pay attention to this disease to arrive at a correct diagnosis.

## Data availability statement

The original contributions presented in the study are included in the article/supplementary material. Further inquiries can be directed to the corresponding author.

## Ethics statement

The studies involving human participants were reviewed and approved by Ethics Committee of Zunyi Medical University. The patients/participants provided their written informed consent to participate in this study. Written informed consent was obtained from the individual(s) for the publication of any potentially identifiable images or data included in this article.

## Author contributions

The study design was performed by BT, XW, and LD. Review of patient data and critical comments were performed by XW, LD, CL, SZ, NT, JH, and BT. XW and LD reviewed and described the pathologic and endoscopic findings. The manuscript was written by XW, LD, and BT. All authors contributed to the article and approved the submitted version.
